# Assessing the association between musculoskeletal injury and mental health in rugby players—A cross-sectional study

**DOI:** 10.1371/journal.pone.0343140

**Published:** 2026-04-29

**Authors:** Mordicai Atinga, Jasmit Shah, Sarah Karanja, Mark Lutomia Lumbasi, Miriam Wambui Ndungu, Sarah Kiburi, James Ondiege, Njalalle Baraza

**Affiliations:** 1 Orthopaedic Section, Aga Khan University Hospital, Nairobi, Kenya; 2 Brain and Mind Institute, Aga Khan University, Nairobi, Kenya; 3 Department of Internal Medicine, Aga Khan University, Nairobi, Kenya; 4 Kenya Medical Research Institute (KEMRI), Nairobi, Kenya; 5 Ministry of Health, Nairobi, Kenya; 6 Kenyatta National Hospital, Nairobi, Kenya; Portugal Football School, Portuguese Football Federation, PORTUGAL

## Abstract

**Background:**

Musculoskeletal injuries are common among athletes and can negatively affect both physical and psychological wellbeing. Beyond physical disability, injuries have been linked to poor mental health, loss of athletic identity, and increased substance use. In low- and middle-income countries (LMICs), where awareness and management of mental health remain limited, the psychological burden of sports injuries may be underrecognized. Rugby, a physically demanding contact sport, has one of the highest rates of injury, but little is known about how such injuries influence mental health and related outcomes in African contexts. This study explored the relationship between musculoskeletal injuries and health outcomes—including mental health, physical functioning, identity, and substance use—among rugby players in Kenya.

**Methodology:**

We conducted a cross-sectional survey between October 2024 and November 2024 among rugby players from ten clubs across Kenya. Eligible participants were adults aged 18 years and above, recruited using convenience sampling. Data were collected through electronic questionnaires that captured demographics and included various psychological and health assessment tools. Summary statistics were presented as medians and interquartile ranges for continuous data and frequencies and percentages for categorical data. The non-parametric Kruskal–Wallis test was used to compare the continuous variables and Fisher’s exact test was used to compare the categorical variables between group associations.

**Results:**

A total of 400 players were recruited and included in the analysis. The median age was 24.0 years (IQR 21.0–27.0) and majority were males (89.8%). Over half (52.5%) reported a significant injury in the past two years, most commonly affecting the knee (41.0%), ankle (32.4%), and shoulder (21.0%). The mean GHQ-12 score was 22.4 (SD = 5.6), indicating some burden of mental health symptoms, and injured players had significantly higher GHQ-12 scores (p = 0.001). On the SF-12, the mean physical and mental component scores were 84.5 (SD = 12.6) and 60.5 (Sd = 8.6), with injured players reporting lower physical scores (p < 0.001). The AIMS shower strong athletic identity overall with injured players demonstrating significantly higher social identity scores (p = 0.007). The ASSIST revealed that 72.8% had ever used substances, with alcohol (71.8%), cannabis (19.5%), and tobacco (13.3%) being the most common; cannabis use was significantly higher among injured players (p = 0.002).

**Conclusion:**

Musculoskeletal injuries among rugby players were associated with poorer mental health, reduced physical functioning, and greater cannabis use. These findings underscore the need for integrated injury management approaches that address both physical rehabilitation and psychological wellbeing, including mental health screening, counselling, and substance use surveillance.

## Introduction

Sport and physical activity are increasingly recognized as powerful determinants of both physical and mental health, contributing not only to disease prevention but also to social and personal development. Regular participation improves cardiovascular health, stamina, and bone strength, while also reducing the risk of chronic diseases later in life [1]. Beyond these psychological benefits, sports-particularly organized and team-based sports, also contributes to social cohesion, discipline, teamwork and a sense of belonging across the life course. Participation in team sports, in particular, offers additional advantages by fostering a strong sense of community, enhanced social integration, and personal growth. These benefits are especially evident among youth, where engagement in sports has been linked to improved social skills [2,3], enhanced personal development [4], and a reduction in substance use [5,6]. Collectively, these factors have also contributed to a higher quality of life [7] and encourage continued participation and long-term commitment to sports [8].

Despite these benefits, sports participation carries the risk of injury, which can significantly impact quality of life. Musculoskeletal injuries, in particular, may impair mobility, cause chronic pain, and disrupt athletes’ daily functioning. In high-contaxt sports, these injuries are especially common an may result in prolonged absence from training and competition. Beyond the physical consequences, injuries can negatively affect psychological well-being. Mainwaring et al. demonstrated that the emotional disturbance following injury varies by injury type, with knee injuries showing greater psychological impact than concussions [9]. Other studies highlight that injury may alter atheles’ peceptions of their bodies, interference with rehabilitation due to perception of the injury, and contribute to reinjury anxiety [10,11], identity loss [12,13], and depressive symptoms [9]. These psychological responses may adversely affect rehabilitation adherence and delay return to play. If unrecognized, these psychological sequelae may prolong recovery and compromise return to play.

In many low- and middle-income countries (LMICs), awareness of mental health conditions remains suboptimal [13]. Stigma persists among both in healthcare workers [14,15] and the wider public [16], contributing to underdiagnosis and undertreatment. Mental health concerns are common in primary care settings in high income countries accounting for 12% of global disease burden, whilst major mental disorders have a 4% prevalence which is comparable with the rate in Kenya [17]. Despite this burden, mental health services in LMICs remain under-resourced, and access to specialized psychological care is limited. As such, the impact of musculoskeletal injuries on athletes’ mental health may be underrecognized and undertreated.

Most existing evidence on the psychological impact of sports-related injury comes from high-income settings and predominantly focuses on Western athlete populations. There is limited empirical research and understanding of how injuries affect mental health among athletes in sub-Saharan Africa (SSA), where cultural perceptions of injury, resilience, and mental health, as well as limited access to mental health services, may alter the experience and recovery trajetectories. Rugby is one of the fastest-growing contact sports in Kenya, with a high risk of musculoskeletal injury, but little is known about the mental health consequences in this group. To date, no studies have specifically examined the association between musculoskeletal injury and mental health outcomes among rugby players in Kenya, representing a critical gap in the literature. This study therefore aimed to assess the relationship between musculoskeletal injuries and mental health outcomes in rugby players in Kenya.

## Materials and methods

We carried out a cross-sectional survey study between 21^st^ October 2024 and 29^th^ November 2024. The study was conducted across ten rugby clubs in Kenya. The clubs were introduced through the Kenya Rugby Union, and those that expressed interest in participation were contacted and included in the study. All adult players aged ≥ 18 years were eligible to participate. Players undergoing treatment for non-sport-related mental health concerns were excluded. Recruitment was conducted at the club level, and participation was dependent on the willingness of individual clubs and players.

Since there is limited data on mental health status of athletes in Kenya, we used a conservative prevalence estimate of 50% (which yields the maximum sample size for a proportion) and a 5% precision level. Based on this, the minimum required sample size was 385.

The survey (study questionnaire) included sociodemographic characteristics (age, gender, education level etc) and validated health assessments tools. All instruments used in this study are standardized, validated, and widely applied, with established reliability in measuring mental health, quality of life, athletic identity, post-traumatic stress symptoms, and substance use. No pilot study was conducted as all tools were previously validated in multiple populations. These tools captured the following key variables:

a) General mental health status, assessed using the general health questionnaire (GHQ-12) [18], a 12-item instrument scored on a 4-point Likert scale (range 12–48), where higher scores indicate greater likelihood of psychological distress. The GHQ-12 has shown strong internal consistency with Cronbach’s alpha values typically ranging from 0.82 to 0.93.
b) Health-related quality of life, measured using the 12-Item Short Form health survey (SF-12) [19], comprising a physical component and mental component, both ranging from 0–100, with scores above 50 indicate above-average health status. The SF-12 demonstrates high construct validity and reliability, with reported Cronbach’s alpha values exceeding 0.80 for both physical and mental components.
c) Athletic identity, assessed using the Athletic Identity Measurement Scale (AIMS) [20], which evaluates three domains—social identity, exclusivity, and negative affectivity. Higher scores indicate stronger identification with the athlete role. The AIMS has shown good internal consistency (Cronbach’s alpha ranging from 0.81 to 0.93) and construct validity in measuring athletic identity.
d) Post-traumatic stress symptoms, screened using PC-PTSD-5 [21], a five-item screen for post-traumatic stress disorder symptoms, with higher scores suggesting greater PTSD risk. The PC-PTSD-5 has demonstrated good sensitivity and specificity in detecting PTSD symptoms in clinical and community settings.
e) Substance use risk, assessed using the Alcohol, Smoking and Substance Involvement Screening Test (ASSIST) [22], which screens for risky substance use, including tobacco, alcohol, cannabis, and other drugs, with higher scores reflecting greater involvement. The ASSIST has been validated by the World Health Organization, showing strong reliability and concurrent validity for identifying substance use risk.

Data were collected using a structured questionnaire administered through the Research Electronic Data Capture (REDCap) platform (Vanderbilt University and National Institute of Health) [23]. Data collection was conducted in person by trained research assistants through one-on-one interactions, allowing for clarification of questions and real-time entry of responses into REDCap. This approach minimized missing data and ensured confidentiality. Written informed consent was obtained from all participants. Players were informed that they could withdraw from the study at any time without any consequences. Approval for this study was obtained from the Institutional Scientific and Ethics Review Committee (ISERC) (Ref:2023/ISERC-78(V8)) at the Aga Khan University, Nairobi and Kenya's National Commission for Science, Technology and Innovation (NACOSTI/P/24/33317). Participants were able to withdraw from the study at any time without consequences.

Continuous data were analyzed using medians and interquartile ranges (IQRs) and means and standard deviations (SD) whereas categorical data were analyzed as frequencies and percentages. The non-parametric Kruskal–Wallis test was used to compare the continuous variables and Fisher’s exact test was used to compare the categorical variables between group associations. Furthermore, logistic regression analyses was conducted to examine the association between the various questionnaires with injury and presented as odds ratio (OR) with 95% confidence intervals (CI). Data analysis was performed using SPSS statistical software V. 20.0 (IBM, Armonk, NY, USA). The significance level was set at α = 0.05, and all tests were two-tailed.

## Results

### Overall characteristics

A total of 400 participants completed the questionnaires. The median age of the players was 24.0 years (IQR: 21.0–27.0) with majority of the players, 89.8% (n = 359) being males. Majority of the participants, 73.8% (n = 295) completed Secondary or Bachelors education, 49.0% (n = 196) were employed or self-employed and 87.5% (n = 350) were single. Only over a half, 57.0% (n = 228) got paid to play. Injury reporting was experienced in 52.5% (n = 210) with majority of the injury reported in knees (41.0%), ankle (32.4%), and shoulders (21.0%) whereas only 38.3% reported having been diagnosed with concussion. Table 1 summarizes the characteristics of the participants.

**Table 1:** Overall demographic characteristics of 400 participants.

Age (years)	(median [IQR])	24.0 [21.0, 27.0]	
Education Completed	Primary	2	0.5%
	Secondary	113	28.3%
	Bachelors	182	45.5%
	Masters	12	3.0%
	Other	91	22.8%
Gender	Male	359	89.8%
	Female	41	10.3%
Employment Status	Self Employed	92	23.0%
	Employed	104	26.0%
	Not Employed	200	50.0%
	Other	4	1.0%
Relationship Status	Single	350	87.5%
	Married	36	9.0%
	Dating	13	3.3%
	Divorced	1	0.3%
Sport	Rugby	400	100.0%
Do you get paid to play?	Yes	228	57.0%
	No	172	43.0%
Time played (years)	(median [IQR])	7.0 [3.0, 11.0]	
Diagnosed with concussion	Yes	153	38.3%
	No	247	61.8%
Experience injury in last 2 years that has stopped you from playing	Yes	210	52.5%
	No	190	47.5%
Body Parts injured	Ankle	68	32.4%
	Knee	86	41.0%
	Hamstring	16	7.6%
	Hip	5	2.4%
	Spine	8	3.8%
	Shoulders	44	21.0%
	Hand/Wrist	26	12.4%
	Other	16	7.6%

### Overall health questionnaires

The mean GHQ-12 score was 22.4 (SD = 5.6), indicating a notable burden of mental health concerns. For the SF-12, mean scores were 84.5 (SD = 12.6) for the physical component and 60.5 (SD = 8.6) for the mental component. On the AIMS, players demonstrated strong athletic identity, with the highest scores in social identity (88.3 ± 11.1), exclusivity (75.8 ± 25.1) and negative affectivity was (87.9 ± 22.3). Substance use was common, with 72.8% reporting lifetime use of at least one substance. Alcohol (71.8%) was the most common, followed by cannabis (19.5%) and tobacco (13.3%). Based on the PC-PTSD-5 screening, 76.5% of participants scored between 0–2 (negative screen), while 23.5% scored between 3–5 (positive screen). Table 2 presents the overall health questionnaire outcomes.

**Table 2:** Overall summaries of the various health questionnaires of 400 participants.

GHQ- 12 Questionnaire	Overall	22.4 (5.6)
SF −12 Questionnaire	Physical Component	84.5 (12.6)
	Mental Component	60.5 (8.6)
AIMS Questionnaire	Social Identity	88.3 (11.1)
	Exclusivity	75.8 (25.1)
	Negative Affectivity	87.9 (22.3)
PTSD Screening	Negative	306 (76.5%)
	Positive	94 (23.5%)
Ever used any substance	Yes	271 (72.7%)
	No	109 (27.3%)
In your life, which of the following substances have you ever used? (NON-MEDICAL USE ONLY)	Tobacco products	53 (13.3%)
	Alcohol beverages	287 (71.8%)
	Cannabis	78 (19.5%)
	Cocaine	2 (0.5%)
	Amphetamine type stimulants	4 (1.0%)
	Inhalants	4 (1.0%)
	Sedatives or sleeping pills	17 (4.3%)
	Hallucinogens	4 (1.0%)
	Opioids	1 (0.3%)

### Associations between injury and health outcomes

Injury status was significantly associated with several health outcomes. Injured players had higher GHQ-12 scores compared to non-injured players (23.4 (SD = 6.2) vs 21.2 (SD = 4.7); p = 0.001), reflecting poorer mental health. SF-12 physical scores were significiantly lower among injured players as compared to non-injured players (81.9 (SD = 14.2) vs 87.4 (SD = 9.9); p < 0.001). SF-12 mental scores were lower among injured players as compared to non-injured players but not statistically significant (60.0 (SD = 8.8) vs 61.1 (SD = 8.4); p = 0.233). On the AIMS questionnaire, injured players demonstrated stronger social identity (89.4 (SD = 10.7) vs 87.0 (SD = 11.4); p = 0.007) and also demonstrated higher scores in exclusivity and negative affectivity but not statistically significant. PTSD screening shower higher positive screening among injured players as compared to non-injured players but not statistically significant (24.8% vs 22.1%; p = 0.557). Regarding substance use, overall use was higher among injured players, but only cannabis use showed a significant association with injury (25.2% vs 13.2%; p = 0.002). Table 3 summarizes the differences for the different health questionnaires with injury.

**Table 3:** Association of various health questionnaires with injury.

		YES INJURY		NO INJURY		P value
		(n = 210)		(n = 190)		
GHQ12 Questionnaire	Total Score	23.4 [6.2]		21.2 [4.7]		0.001
SF12 Questionnaire	Physical Health Total Score	81.9 [14.2]		87.4 [9.9]		<0.001
	Mental Health Total Score	60.0 [8.8]		61.1 [8.4]		0.233
AIMS Questionnaire	Social Identity	89.4 [10.7]		87.0 [11.4]		0.007
	Exclusivity	76.6 [25.1]		75.0 [25.1]		0.474
	Negative Affectivity	88.0 [22.4]		87.8 [22.3]		0.997
Ever used any substance	Yes	159	75.7%	132	69.5%	0.178
	No	51	24.3%	58	30.5%	
PTSD Screening	Negative	158	75.2%	148	77.9%	0.557
	Positive	52	24.8%	42	22.1%	
In your life, which of the following substances have you ever used? *(NON-MEDICAL USE ONLY)*	Tobacco products	34	16.2%	19	10.0%	0.077
	Alcohol beverages	157	74.8%	130	68.4%	0.182
	Cannabis	53	25.2%	25	13.2%	0.002
	Cocaine	2	1.0%	0	0.0%	0.500
	Amphetamine type stimulants	2	1.0%	2	1.1%	1.000
	Inhalants	3	1.4%	1	0.5%	0.625
	Sedatives or sleeping pills	11	5.2%	6	3.2%	0.332
	Hallucinogens	4	1.9%	0	0.0%	0.125
	Opiods	1	0.5%	0	0.0%	1.000

*Tests used for categorical data – Fishers exact test.

*Tests used for continuous data – Kruskal Wallis test.

In the logistic regression analysis, higher GHQ12 overall scores were significantly associated with increased odds of injury in both the univariable (OR = 1.07, 95% CI: 1.03–1.11, p < 0.001) and multivariable models (AOR = 1.08, 95% CI: 1.04–1.12, p < 0.001). In contrast, higher SF12 physical health scores demonstrated a protective effect, with reduced odds of injury observed in both univariable (OR = 0.96, 95% CI: 0.95–0.98, p < 0.001) and multivariable analyses (AOR = 0.96, 95% CI: 0.94–0.98, p < 0.001). Table 4 summarizes the logistic regression results for the different health questionnaires with injury.

**Table 4:** Regression analyses examining factors associated with injury.

	OR (95% CI)	P value	AOR (95% CI)	P value
GHQ12 Overall Score	1.07 [1.03, 1.11]	<0.001	1.08 [1.04, 1.12]	<0.001
SF12 Physical Health Total Score	0.96 [0.95, 0.98]	<0.001	0.96 [0.94, 0.98]	<0.001
SF12 Mental Health Total Score	0.99 [0.96, 1.01]	0.224	0.99 [0.97, 1.01]	0.292
AIMS – Social Identity	1.02 [1.00, 1.04]	0.027	1.02 [1.00, 1.04]	0.018
AIMS – Exclusivity	1.00 [0.99, 1.01]	0.519	1.01 [1.00, 1.01]	0.179
AIMS – Negative Affectivity	1.00 [0.99, 1.01]	0.950	1.00 [0.99, 1.01]	0.721
Ever used substance use	1.37 [0.88, 2.13]	0.162	1.14 [0.71, 1.83]	0.588
PTSD – Positive Screen	1.16 [0.73, 1.84]	0.532	1.30 [0.80, 2.09]	0.288

*OR: Odds Ratio; CI: Confidence intervals; AOR: Adjusted Odds Ratio.

*Adjusted for gender, education, employment and relationship.

## Discussion

This study primarily aimed to explore and understand the relationship between musculoskeletal injuries and the mental health of rugby players in Kenya. The GHQ-12 revealed significantly higher scores among injured players compared to non-injured players, highlighting the potential for injury to negatively impact mental health. These findings are consistent with Kilic et al. [24] and Rogers et al. [25], who demonstrated increased symptoms of anxiety and depression in injured athletes. In contrast, Du Preez et al. found no difference in the mental health symptoms of elite rugby players who had suffered injury compared to uninjured players [26]. The discrepancies across studies may reflect methodological differences, timing of assessment, or protective factors unique to different athletes. Other works, including Brewer et al. [27] and Putukian [28] have emphasized the variable psychological responses to injury, particularly when mental health is assessed cross-sectionally rather than longitudinally.

Substance use emerged as another important theme. In this study 72.8% reported having used a substance. The most prevalent substance was alcohol, followed by cannabis and tobacco. While overall substance use was not significantly associated with injury, cannabis use was significantly higher among injured players. This trend may suggest that athletes may turn to substance use as a coping mechanism to manage pain, psychological distress, or the loss of athletic engagement. Similar patterns have been observed by Hume et al. [29] who found higher alcohol consumption among injured athletes but no difference in mental health symptoms and Kasper et al. [30] found in rugby players, 18% had used cannabidiols and 8% were still using and some of the main reasons were pain management and improved sleep quality. Further more, in a systematic review by Docter et al. found that 23.4% reported use of cannabis across a variety of sports, countries and age groups in the 1 year period and was a higher prevalence than in non-sporting populations [31]. Some of the stated reasons were sleep improvement, pain management and aiding relaxation. Reardon et al. reported higher use of cannabis in rugby and a number of other contact and adrenaline sports as compared to other sports [32].

Demographic and socioeconomic variables—including employment status, relationship status, and playing status—were not significantly associated with differences in mental health among injured and non-injured players. In the integrated model of psychological response to sports injury and rehabilitation, demographic factors are considered an important factor determining how athletes address injury [33]. This is in contrast to Mitchell et al. who demonstrated the buffering effect of a support network in injured athletes in avoiding mental health deterioration particularly with regard to isolation, restlessness and feeling cheated or their body had failed them [34].

Players demonstrated high levels of identity across social identity, exclusivity, and negative affectivity. Injured athletes scored higher in social identity, but exclusivity and negative affectivity did not differ significantly between groups. These findings differ from studies such as Renton et al. [35] and Brewer et al [27,36], which have demonstrated stronger associations between injury and disruptions in athletic identity. It is possible that the strong cultural identity linked to rugby in Kenya may buffer against some of these changes. Rugby culture fosters a deep sense of belonging, and the disruption caused by injury can lead to identity crises and feelings of social disconnection. This psychological shift may exacerbate mental health symptoms and increase vulnerability to maladaptive coping strategies such as substance use.

No significant differences were found in post-traumatic stress symptoms, as measured by the PC-PTSD-5. This contrasts with findings from Bateman et al [37], Aron et al. [38], and Brassil and Salvatore [39], who identified higher PTSD symptomatology in athletes following injury or concussion. The absence of differences in this study may reflect underreporting, recall bias, or resilience factors not captured in the current design.

This study has several limitations. Selection bias may have been introduced by including only clubs that expressed interest and responded to the initial invitation. The absence of resilience scoring may have limited understanding of psychological variability between injured and non-injured players. Reliance on self-reported concussion history and mental health symptoms raises the possibility of recall bias. Finally, the cross-sectional design precludes assessment of changes over time; longitudinal studies are needed to better characterize the trajectory of mental health outcomes following injury.

Despite these limitations, the study had notable strengths. It achieved a large sample size with high response rates Data collection was comprehensive, covering injury history, mental health, substance use, and athletic identity. The inclusion of multiple psychological and behavioural measures provided a nuanced understanding of the mental health impacts of musculoskeletal injury in rugby players.

## Conclusion

Taken together, these findings emphasize the necessity for holistic injury management programs that integrate mental health screening, social support mechanisms, and substance use surveillance. Future research should explore targeted psychological interventions that help athletes maintain their identity and well-being during rehabilitation. Strengthening support networks and promoting adaptive coping strategies may mitigate the adverse mental health and behavioural outcomes observed in injured athletes. Further research into the effect on injuries in women as we had a small number of female participants.

## Supporting information

S1 Table Anonymous data supporting the results. (XLS)
